# Stacked CT radiomics, deep learning and clinical feature models for differentiating benign and malignant solitary pulmonary nodules

**DOI:** 10.1038/s41598-026-49720-8

**Published:** 2026-04-28

**Authors:** Jie Zong, Biyun Jiang, Haiyan Li, Zhiling Li

**Affiliations:** 1 Department of Radiology, Huabei Petroleum Administration Bureau General Hospital, Renqiu City, Cangzhou, 062552 Hebei Province People’s Republic of China; 2 Central Laboratory, Huabei Petroleum Administration Bureau General Hospital, Renqiu, Hebei Province China; 3 Department of Radiology, Qianxi County People’s Hospital, Qianxi, Hebei Province China; 4 Department of general surgery, Huabei Petroleum Administration Bureau General Hospital, Renqiu, Hebei Province China

**Keywords:** Solitary pulmonary nodule, Probability stacking, Radiomics, Deep learning, Serological biomarkers, Cancer, Computational biology and bioinformatics, Diseases, Medical research, Oncology

## Abstract

Solitary pulmonary nodules (SPNs) are common incidental findings on high-resolution CT, and differentiating benign from malignant lesions still depends mainly on invasive biopsy, highlighting the need for accurate non-invasive tools. In this retrospective multicenter study we analyzed 201 SPN patients (110 malignant, 91 benign) from three hospitals (2020–2024). We developed three single-modality classifiers—a clinical model incorporating demographic data, medical history, imaging characteristics, and seven serum tumor-associated autoantibodies, a radiomics model based on 17 LASSO-selected CT features extracted from manually segmented volumes of interest, and a deep learning visual classifier based on a modified Swin-Transformer architecture—and then fused their per-patient malignancy probabilities with a stacked ensemble using five meta-learners. The integrated model achieved an AUC of 0.936 (95% CI: 0.895–0.971) in the training set and 0.823 (95% CI: 0.699–0.934) in the independent test set, with 80.3% accuracy, 81.6% precision, a recall of 79.4%, and an F1-score of 0.797. Decision curve analysis showed that the fusion model had high clinical net benefit. Multimodal stacking of clinical, radiomic, deep-learning and serological information significantly enhances SPN risk stratification beyond single-modality approaches and may reduce unnecessary invasive procedures in routine practice.

**Supplementary Information:**

The online version contains supplementary material available at 10.1038/s41598-026-49720-8.

## Introduction

A solitary pulmonary nodule (SPN) refers to a focal, non-translucent lesion with a diameter less than 30 mm, fully surrounded by aerated lung parenchyma^1^. Differentiating benign from malignant SPNs remains a significant clinical challenge^2^. With the widespread use of high-resolution computed tomography (HRCT), detection rates have increased substantially^3^. Population-based screening identifies SPNs in 2% to 24% of individuals^4^, and the probability of malignancy may exceed 70% in certain subgroups, depending on demographic characteristics and nodule morphology^5^. Currently, definitive histopathological diagnosis still relies on invasive procedures such as percutaneous biopsy or surgical resection^6^, which are associated with potential complications^7^ and may result in unnecessary treatment of benign nodules^8^.

Traditional CT-based diagnosis is affected by inter-observer variability due to the subjective nature of visual interpretation^9^. Recent advances in artificial intelligence have enabled quantitative analysis and provided new possibilities for non-invasive evaluation^10,11^. Radiomics-based machine learning models have reported areas under the curve (AUCs) ranging from 0.82 to 0.91^12,13^; deep-learning visual-classification networks have achieved accuracies exceeding 90%; and multimodal PET/CT analyses have shown inter-reader agreement rates as high as 97%. However, most current approaches rely exclusively on imaging features, insufficiently incorporate serum biomarkers such as autoantibodies, and frequently lack external validation, adequate sample sizes, and model interpretability^14^.

To overcome these limitations, we propose a novel evidence-based framework that bridges the diagnostic gap between molecular biomarkers and clinical imaging. Specifically, the novelty of this study lies in the synergistic integration of systemic biological immune signatures and multi-scale radiological data, built upon three key technical pillars: (1) Bio-Radiological Multimodal Fusion: integrating four heterogeneous dimensions (semantic clinical factors, seven lung cancer-associated serum autoantibodies, handcrafted radiomics, and deep-learning visual representations) to capture both the host’s systemic immune response and the nodule’s localized morphological patterns; (2) Specialized Architecture for Small Lesions: introducing a customized Swin-Transformer^15^ architecture equipped with FusionCNN^16^ and QJBlock^17^ modules to effectively extract features from small and indeterminate nodules; and (3) Meta-Learning via Stacked-Ensemble: utilizing a meta-learner strategy to dynamically weigh diagnostic probabilities across modalities, thereby mitigating the “curse of dimensionality” and ensuring superior generalizability.

By addressing the limitations of single-modality analysis, the model enhances the differentiation between benign and malignant SPNs. The study aims were as follows: (1) to construct and validate three unimodal predictive models; (2) to develop an optimized stacked-ensemble model that integrates multi-source data; and (3) to systematically evaluate diagnostic performance, calibration, and clinical utility across multicenter independent cohorts, with the goal of providing a personalized decision-support tool for SPN diagnosis.

## Methods

### Materials and methods

#### Study population

This retrospective multicenter study and all experimental protocols were approved by the Clinical Trial Ethics Committee of Huabei Petroleum Administration Bureau General Hospital (Approval No. EC-2023-YuanNeiKeYan-03). We confirm that all methods were carried out in accordance with relevant guidelines and regulations, including the 1964 Declaration of Helsinki and its later amendments. Given the retrospective nature of the study, the requirement for written informed consent was waived by the aforementioned Ethics Committee. A total of 201 patients were consecutively enrolled between January 2020 and August 2024 from three centers with independent physician teams and patient populations: Huayou Geriatric Hospital (Center 1, *n* = 13), Huabei Petroleum Construction Hospital (Center 2, *n* = 69), and Huabei Petroleum Administration General Hospital (Center 3, *n* = 119). Inclusion criteria were as follows: (i) chest CT demonstrating a solitary pulmonary nodule (SPN) with a maximum diameter ≤ 30 mm; (ii) histopathological confirmation or a minimum of 36 months of clinical follow-up; and (iii) availability of pre-operative serum testing for seven tumor-associated autoantibodies (TAABs). Patients were excluded if (i)clinical data were incomplete or (ii) imaging artifacts significantly impaired diagnostic quality.

#### CT image acquisition and preprocessing

Chest CT scans were performed using four different scanners: Revolution CT and LightSpeed VCT (GE Healthcare, Chicago, IL, USA), Alexion CT (Toshiba Medical Systems, Ōtawara, Japan), and uCT 550 (United Imaging Healthcare, Shanghai, China). To minimize source-level variability, all examinations were acquired with a tube voltage of 120 kVp and reconstructed using a uniform Lung Reconstruction Algorithm. The reconstruction slice thickness and increment ranged from 0.625 to 1.25 mm, with a matrix size of 512 × 512. Prior to feature extraction, voxel-level 2D Intensity Background Standardization and Normalization were performed using RIAS (Radiomics Image Analysis Software, https://github.com/lisherlock/RMIT) Version 1.0.0 to harmonize Hounsfield Unit (HU) distributions across varied slice thicknesses and scanner vendors^18^. Detailed scanning parameters are presented in the Supplementary Table S1.

#### Clinical data collection and baseline characteristics

Comprehensive clinical data were collected for each patient, including smoking status, alcohol consumption, family history of malignancy, hypertension, diabetes mellitus, hyperlipidemia, history of occupational dust exposure, previous tumors, history of pulmonary disease, body mass index (BMI), surgical approach, tumor characteristics, pathological subtype, and peripheral blood counts. The serum levels of seven tumor-associated autoantibodies (TAABs)—p53, PGP9.5, SOX2, GAGE 7, GBU4-5, MAGE A1, and CAGE—were measured using commercial ELISA kits (Hangzhou KaibaoLuo Biotechnology Co., Ltd., Hangzhou, China) on the BIOTEK SYNERGY HT platform. The positive cut-off values were established based on a large-scale multicenter validation study (*n* = 2,008), where thresholds were calculated as the Mean + 4 Standard Deviations (SD) of healthy control concentrations. Specifically, the cut-off values were: p53 (13.1 U/mL), PGP9.5 (11.1 U/mL), SOX2 (10.3 U/mL), GAGE 7 (14.4 U/mL), GBU4-5 (7.0 U/mL), MAGE A1 (11.9 U/mL), and CAGE (7.2 U/mL). To ensure assay standardization, all samples were processed in a single centralized laboratory by technicians blinded to the clinical and pathological data. Imaging features were systematically recorded, including nodule location; morphological attributes and perinodular characteristics (tumor–lung interface, vascular changes, bronchial features, and pleural involvement).

#### Clinical data modelling

To ensure a balanced distribution of class labels and preserve multi-center heterogeneity, the entire dataset was partitioned into a training cohort and an independent testing cohort in a 7:3 ratio using **stratified random sampling** (stratified by pathological labels) via the scikit-learn library in Python. This unified split framework successfully maintained consistent malignancy rates (55.0% vs. 54.1%) and representative center distributions between the two cohorts. Crucially, this identical patient allocation was strictly enforced across all subsequent modeling phases (clinical, radiomics, and deep learning) to facilitate accurate multi-modal ensembling without data leakage. All clinical variables were standardized using z-score normalization. Normality was assessed using the Shapiro-Wilk test; variables with a normal distribution were analyzed using Student’s *t*-test, while non-normally distributed variables were assessed using the Mann–Whitney *U*-test to identify statistically significant features. Significant variables were further refined through a sequential feature selection pipeline involving recursive feature elimination (RFE) and the least absolute shrinkage and selection operator (LASSO). The optimal regularization parameter ($\:\alpha\:$) for LASSO was determined as 0.0107 via 10-fold cross-validation (LassoCV). The selected clinical predictors were then used to construct a logistic regression (LR) classifier with ℓ_2_ regularization. The performance metrics of the clinical model are detailed in Table 2.

### Radiomics modelling

#### Image segmentation

Thin-slice CT images were retrieved from the picture archiving and communication system (PACS) and converted in Digital Imaging and Communications in Medicine (DICOM) format. All images were imported into ITK-SNAP (version 4.2.0, www.itksnap.org) for manual segmentation of three-dimensional (3D) regions of interest (ROIs). Specifically, the entire volume of each nodule was contoured slice-by-slice on axial images by two independent board-certified radiologists in a random sequence to evaluate inter-observer reliability. The resulting segmentation masks were saved separately for subsequent feature extraction and consistency analysis.

#### Radiomic feature extraction

Radiomic features were extracted from the segmented 3D ROIs using the IBSI-compliant RIAS (version 1.0.0, https://github.com/lisherlock/RMIT). Prior to extraction, all images were resampled to an isotropic voxel size of 1.0 × 1.0 × 1.0 mm³ to ensure spatial consistency. To enhance the feature space and capture high-dimensional information, several image transformations were applied, including Wavelet, Laplacian of Gaussian (LoG), Square, and Exponential filters. A total of 1,427 features were extracted, comprising 17 3D-shape features, 285 first-order statistics, and 1,125 texture-based features (subdivided into 360 Gray-level co-occurrence matrix [GLCM], 240 Gray-level run-length matrix [GLRLM], 240 Gray-level size-zone matrix [GLSZM], 210 Gray-level dependence matrix [GLDM], and 75 neighborhood gray-tone difference matrix [NGTDM] features). A detailed breakdown of these features is provided in Table 1.

**Table 1:** Distribution of the 1,427 extracted radiomic features across different subtypes.

Feature Category	Number of Features	Description
Shape features (3D)	17	Describing the morphological characteristics (e.g., Volume, Surface Area, Sphericity).
First-order statistics	285	Quantifying the distribution of voxel intensities within the ROI (e.g., Mean, Entropy, Skewness).
Gray-level co-occurrence matrix (GLCM)	360	Quantifying the spatial relationships and local texture patterns of pixel pairs.
Gray-level run length matrix (GLRLM)	240	Quantifying the length of consecutive pixels with the same intensity (coarseness).
Gray-level size zone matrix (GLSZM)	240	Quantifying the size of connected regions with identical grey levels.
Gray-level dependence matrix (GLDM)	210	Describing the dependency of pixel intensities within the neighborhood.
Neighboring gray difference matrix (NGTDM)	75	Quantifying the difference between a voxel and its neighbors (Contrast, Complexity).
Total Features	1427	

### Model construction

#### Radiomics classifier

Inter-observer reproducibility between the two radiologists’ segmentations was assessed by calculating the intraclass correlation coefficient (ICC) for each feature. Features with ICC values > 0.75 were retained for further analysis. Adhering to the identical stratified split framework established in the clinical phase, all retained features were standardized using z-score normalization to further minimize institutional batch effects. To identify a stable radiomic signature, a sequential dimensionality reduction strategy was implemented. Initially, the Mann-Whitney U test was used to filter non-significant features (*p* > 0.05), followed by Recursive Feature Elimination (RFE) to retain the top 30 variables. Finally, LASSO regression was applied to select the most predictive features. The regularization parameter ($\:\alpha\:$) was optimized via 10-fold cross-validation, yielding an optimal value of 0.0107. This process resulted in 17 robust features with non-zero coefficients. The selected features were then used to construct a logistic regression (LR) classifier.

#### Deep-learning visual classification

**Fig. 1:**
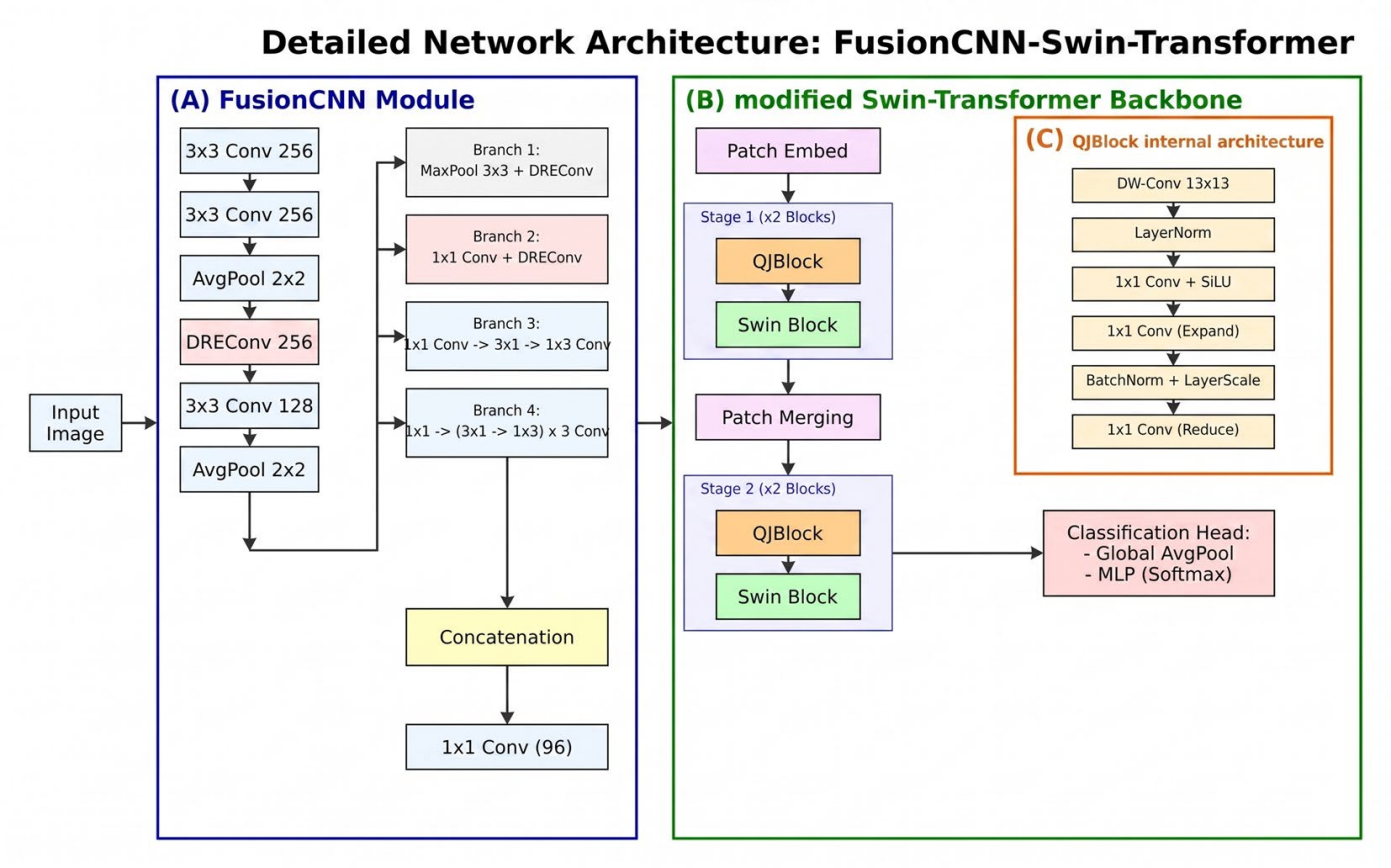
Overall architecture of the proposed FusionCNN-Swin-Transformer network. The model consists of three primary components: (**A**) the FusionCNN module, (**B**) the modified Swin-Transformer backbone, and (**C**) the QJBlock internal architecture. (**A**) FusionCNN Module: A multi-scale feature extraction stage that employs a stem of 3 × 3 convolutions followed by four parallel branches. Specifically, Branches 3 and 4 utilize asymmetric convolutions (3 × 1 and 1 × 3) to capture elongated morphological features while reducing computational overhead. DREConv is integrated to refine features through gated residual paths. (**B**) Swin-Transformer Backbone: The backbone is organized into stages, where each stage includes a QJBlock followed by a standard Swin-Transformer block. Patch merging is applied between stages to reduce spatial resolution and increase channel depth. (**C**) QJBlock: An innovative block designed to mimic global attention via 13 × 13 large-kernel depth-wise convolutions. It incorporates LayerScale and BatchNorm to ensure training stability and enhance the representation of long-range spatial dependencies. The final classification head utilizes adaptive average pooling and a multi-layer perceptron (MLP) with a softmax function to predict the malignancy of pulmonary nodules.

A lung nodule classifier was developed based on a modified Swin-Transformer architecture^15^. To maintain the integrity of the ensemble strategy, the image dataset was partitioned strictly according to the patient-level stratified split previously described. The overall architecture of the proposed FusionCNN-Swin-Transformer network is illustrated in Fig. 1. Two model types were constructed using CT data: a 2D model, based on the axial slice with the largest nodule diameter, and a 2.5D model, combining the axial, sagittal, and coronal slices with the largest diameters.

To reduce computational complexity, the original Swin stages were reduced to two, each containing two Swin blocks, and were replaced by a FusionCNN module. Drawing inspiration from recent advances in asymmetric residual networks^16^, the FusionCNN employs parallel 3 × 1 and 1 × 3 convolutions to capture elongated morphological features while minimizing computational overhead. A QJBlock utilizing 13 × 13 large-kernel depth-wise convolutions^17^ was incorporated into the module to address the limited receptive field of local sliding windows and to enhance global contextual information for small nodules. Additionally, a DREConv layer was introduced to improve feature-extraction capability, leveraging soft attention gating mechanisms to refine salient regional features^19^. Specifically, the deep-learning model was optimized using the AdamW optimizer (learning rate: 1 × 10^− 4^, weight decay: 0.05) and Cross-Entropy Loss. Training lasted for 10 epochs with a batch size of 8. The final model weights were selected based on the peak performance (AUC and F1-score) on the validation set. Twelve model variants were evaluated—six 2D and six 2.5D—based on different combinations of window settings (WW = 1000 / WL = -700; WW = 1500 / WL = -700) and the presence or absence of image normalization. Diagnostic performance was compared across all configurations.

### Stacked-ensemble model

For each patient, the predicted probabilities of malignancy (class = 1) generated by the clinical, radiomics, and deep-learning models were integrated as a new set of three features. These were used to construct a meta-dataset for second-level classification. Five machine learning algorithms—support vector machine (SVM), logistic regression (LR), random forest (RF), Gaussian Naïve Bayes (GNB), and extreme gradient boosting (XGB)—were systematically evaluated. Hyperparameters for these meta-learners were fine-tuned via GridSearchCV with 5-fold cross-validation to achieve the most stable and robust fusion result. Model performance was evaluated using the area under the receiver operating characteristic curve (AUC), accuracy, recall, and F1 score to determine the optimal ensemble classifier. All key implementation details and training hyperparameters for the ensemble stage are summarized in Table 2 to ensure full reproducibility.

**Table 2:** Detailed Summary of Training Hyperparameters and Implementation Platforms.

Phase	Category	Parameter	Value / Description	Implementation
Phase I: Base Models	Deep Learning	Optimizer / Loss	AdamW / Cross-Entropy	PyTorch
		Learning Rate (LR)	1 × 10^− 4^	PyTorch
		Batch Size / Weight Decay	8 / 0.05	PyTorch
		Training Epochs	10 (with pre-trained weights)	PyTorch
	Radiomics & Clinical	Normalization	Z-score standardization	Scikit-learn
		Feature Selection	U-test + RFE + 10-fold LassoCV	Scikit-learn
		Classifier	Logistic Regression ($\:L2$​ penalty)	Scikit-learn
Phase II: Ensemble	Stacked Model	Meta-Learners	LR, SVM, RF, XGB	Scikit-learn
		LR Configuration	Penalty=$\:L2$​, Solver=liblinear	Scikit-learn
		Optimization	GridSearchCV (5-fold CV)	Scikit-learn
		SVM Search Space	$\:C$:2^− 5^∼2^5^; γ:2^− 5^∼2^1^	Scikit-learn

### Performance evaluation and statistical analysis

Continuous variables were reported as mean ± standard deviation (SD) or median with the interquartile range (IQR), while categorical variables were summarized as frequencies and percentages. Group comparisons for continuous variables were performed using the independent-sample *t*-test or the Mann–Whitney *U*-test, and categorical variables were compared using the Chi-square test. Model performance was quantitatively assessed using receiver operating characteristic (ROC) curves, the area under the curve (AUC) with 95% confidence intervals (CIs), accuracy, sensitivity, specificity, and F1-score. Calibration plots and decision curve analysis (DCA) were employed to evaluate model calibration and clinical utility, respectively. All statistical analyses were performed using SPSS (version 27.0; IBM Corp., Armonk, NY, USA), and model construction was conducted using Python (version 3.12).

## Results

**Fig. 2:**
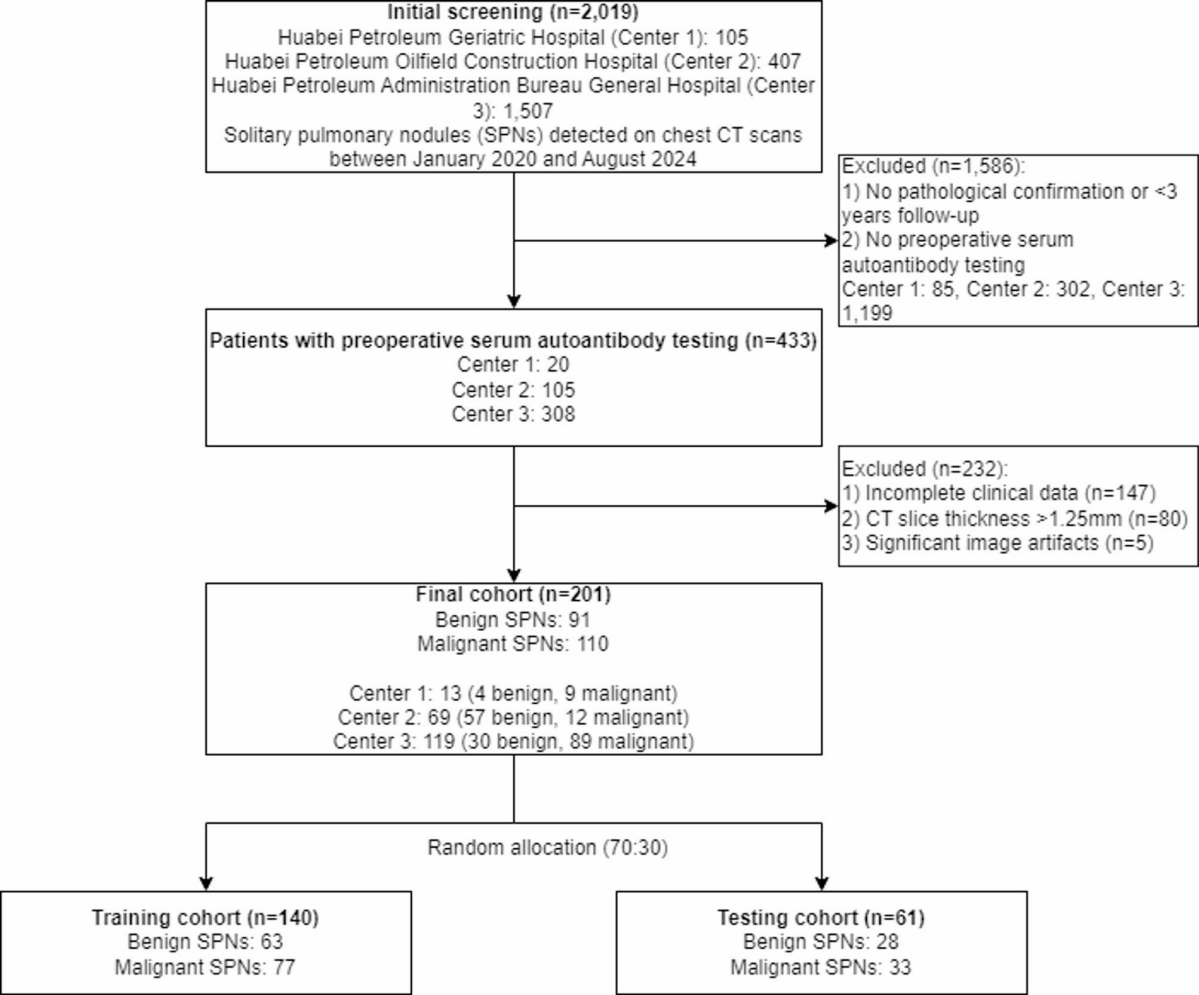
Flowchart of patient enrolment and SPN screening.

Between January 2020 and August 2024, chest CT scans conducted at the three participating centers identified a total of 2,019 patients with solitary pulmonary nodules (SPNs), including 105 from Center 1, 407 from Center 2, and 1,507 from Center 3. After applying the inclusion and exclusion criteria, 201 patients were deemed eligible for analysis, comprising 91 with benign SPNs and 110 with malignant SPNs. These patients were randomly assigned to a training cohort (*n* = 140; 63 benign, 77 malignant) and a testing cohort (*n* = 61; 28 benign, 33 malignant). The patient selection process across the three centers is illustrated in Fig. 2.

### Overall clinical and baseline characteristics

Among the 201 included patients, 91 had benign nodules (Center 1: *n* = 4; Center 2: *n* = 57; Center 3: *n* = 30) and 110 had malignant nodules (Center 1: *n* = 9; Center 2: *n* = 12; Center 3: *n* = 89). A summary of the baseline demographic and clinical characteristics is provided in Table 3.

**Table 3:** Baseline table.

Characteristics	Testing cohort(*n* = 61)	Training cohort(*n* = 140)	*P*-value
Group			0.906
Malignant	28(45.9%)	63(45.0%)	
Benign	33(54.1%)	77(55.0%)	
Gender			0.556
Female	28(45.9%)	58(41.4%)	
Male	33(54.1%)	82(58.6%)	
Age(years)	57.9 ± 11.9	57.0 ± 12.6	0.618
BMI	27.01 ± 3.16	​27.47 ± 3.19	0.346
Smoking History			0.459
No	45(73.8%)	96(68.6%)	
Yes	16(26.2%)	44(31.4%)	
Alcohol History			0.383
No	50(82.0%)	107(76.4%)	
Yes	11(18.0%)	33(23.6%)	
Family History Tumor			0.168
No	59(96.7%)	139(99.3%)	
Yes	2(3.3%)	1(0.7%)	
Hypertension History			0.833
No	38(62.3%)	85(60.7%)	
Yes	23(37.7%)	55(39.3%)	
Diabetes			0.931
No	52(85.2%)	120(85.7%)	
Yes	9(14.8%)	20(14.3%)	
Coronary Heart Disease (CHD)			0.740
No	55(90.2%)	124(88.6%)	
Yes	6(9.8%)	16(11.4%)	
Hyperlipidemia			0.166
No	56(91.8%)	135(96.4%)	
Yes	5(8.2%)	5(3.6%)	
Dust Exposure History			/
No	61(100.0%)	140(100.0%)	
Yes	0(0.0%)	0(0.0%)	
Tumor History			0.465
No	59(96.7%)	132(94.3%)	
Yes	2(3.3%)	8(5.7%)	
Lung Disease History			0.655
No	57(93.4%)	133(95.0%)	
Yes	4(6.6%)	7(5.0%)	
Lung_Lobe			0.728
RUL	21(34.4%)	44(31.4%)	
RML	7(11.5%)	15(10.7%)	
RLL	14(23.0%)	34(24.3%)	
LUL	12(19.7%)	21(15.0%)	
LLL	7(11.4%)	26(18.6%)	
Shape			0.901
Irregular	35(57.4%)	79(56.4%)	
Regular	26(42.6%)	61(43.6%)	
Max Diameter (mm)	10.50 (7.25, 17.50)	9.75 (7.50, 18.00)	0.840
Volume (mm³)	565.08 (198.93, 1891.32)	482.02 (232.06, 1496.59)	0.734
Density			0.593
Subsolid nodule	33(54.1%)	70(50.0%)	
Solid nodule	28(45.9%)	70(50.0%)	
Mean Value	​-186.7 ± 289.5	​​-191.5 ± 283.7	0.915
Cavity Sign			0.627
No	51(83.6%)	113(80.7%)	
Yes	10(16.4%)	27(19.3%)	
Calcification Sign			0.628
No	57(93.4%)	128(91.4%)	
Yes	4(6.6%)	12(8.6%)	
Fat Sign			0.199
No	60(98.4%)	132(94.3%)	
Yes	1(1.6%)	8(5.7%)	
Nodule Lung Interface			0.417
Blurred	9(14.8%)	15(10.7%)	
Clear	52(85.2%)	125(89.3%)	
Spicule Sign			0.801
No	51(83.6%)	115(82.1%)	
Yes	10(16.4%)	25(17.9%)	
Lobulation Sign			0.961
No	22(36.1%)	51(36.4%)	
Yes	39(63.9%)	89(63.6%)	
Spine Sign			0.087
No	41(67.2%)	110(78.6%)	
Yes	20(32.8%)	30(21.4%)	
Air Sign			0.717
No	52(85.2%)	122(87.1%)	
Yes	9(14.8%)	18(12.9%)	
Bronchial Wall Thickening			0.980
No	58(95.1%)	133(95.0%)	
Yes	3(4.9%)	7(5.0%)	
Bronchial Cut Off			0.291
No	55(90.2%)	132(94.3%)	
Yes	6(9.8%)	8(5.7%)	
Vascular Congestion Sign			0.245
No	39(63.9%)	101(72.1%)	
Yes	22(36.1%)	39(27.9%)	
Vascular Attachment Sign			0.835
No	50(82.0%)	113(80.7%)	
Yes	11(18.0%)	27(19.3%)	
Vascular Bundle Sign			0.308
No	31(50.8%)	82(58.6%)	
Yes	30(49.2%)	58(41.4%)	
Vascular Embedding Sign			0.255
No	41(67.2%)	105(75.0%)	
Yes	20(32.8%)	35(25.0%)	
Pleural Thickening			0.737
No	59(96.7%)	134(95.7%)	
Yes	2(3.3%)	6(4.3%)	
Pleural Indentation Sign			0.283
No	48(78.7%)	100(71.4%)	
Yes	13(21.3%)	40(28.6%)	
Pleural Infiltration			0.508
No	61(100.0%)	139(99.3%)	
Yes	0(0.0%)	1(0.7%)	
White_Blood_Cell_Count (WBC)	5.90 ± 2.13	5.90 ± 1.80	0.997
Neutrophil_Count	3.42 ± 1.82	3.37 ± 1.26	0.835
Lymphocyte_Count	​1.88 ± 0.60	1.93 ± 0.75	0.587
Platelet_Count (PLT)	225.8 ± 56.3	239.5 ± 70.0	0.144
P53	1.00 (0.10, 3.65)	0.60 (0.20, 2.20)	0.538
PGP9.5	0.10 (0.10, 0.90)	0.10 (0.10, 0.70)	0.818
SOX2	1.10 (0.15, 3.35)	1.00 (0.10, 2.68)	0.709
GAGE7	2.10 (0.60, 3.70)	1.55 (0.63, 3.43)	0.697
GBU4-5	0.40 (0.10, 4.50)	0.45 (0.10, 3.50)	0.899
MAGE A1	0.10 (0.10, 0.70)	0.10 (0.10, 0.50)	0.725
CAGE	0.10 (0.10, 0.10)	0.10 (0.10, 0.10)	0.220

Continuous variables are presented as mean ± standard deviation (SD) or median (interquartile range, IQR), and categorical variables as n (%). *P*-values were calculated using Student’s t-test for normally distributed continuous variables, the Mann-Whitney U-test for non-normally distributed variables, and the Chi-square test for categorical variables.

Surgical interventions included segmentectomy (*n* = 82), lobectomy (*n* = 40), wedge resection (*n* = 42), and percutaneous core biopsy (*n* = 27). Additionally, ten patients received anti-infective treatment followed by clinical surveillance for 3–5 years.

### Clinical-model performance

**Fig. 3:**
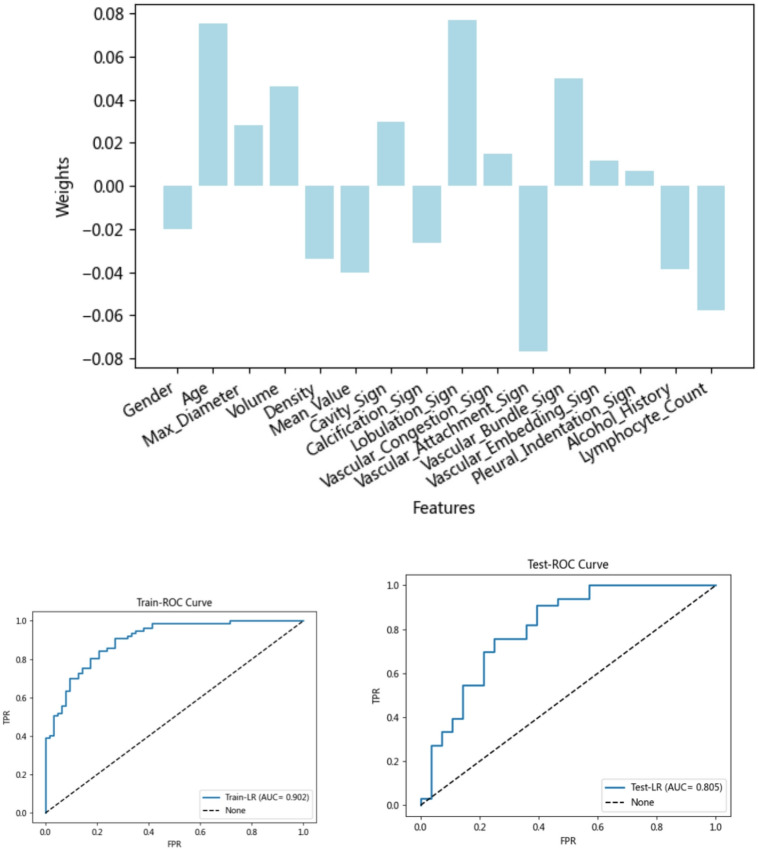
Retained clinical predictors and ROC curves for the clinical model.

The clinical model demonstrated strong discriminatory ability between malignant and benign SPNs, achieving an AUC of 0.902 (95% CI, 0.848–0.947) in the training cohort and 0.805 (95% CI, 0.688–0.915) in the testing cohort. Notably, the lower bounds of the 95% CIs for both cohorts remained significantly above the random-chance threshold of 0.5, confirming the statistical significance of the diagnostic performance. The clinical predictors retained in the final model, along with the corresponding ROC curves, are shown in Fig. 3.

### Radiomics-model results

**Fig. 4:**
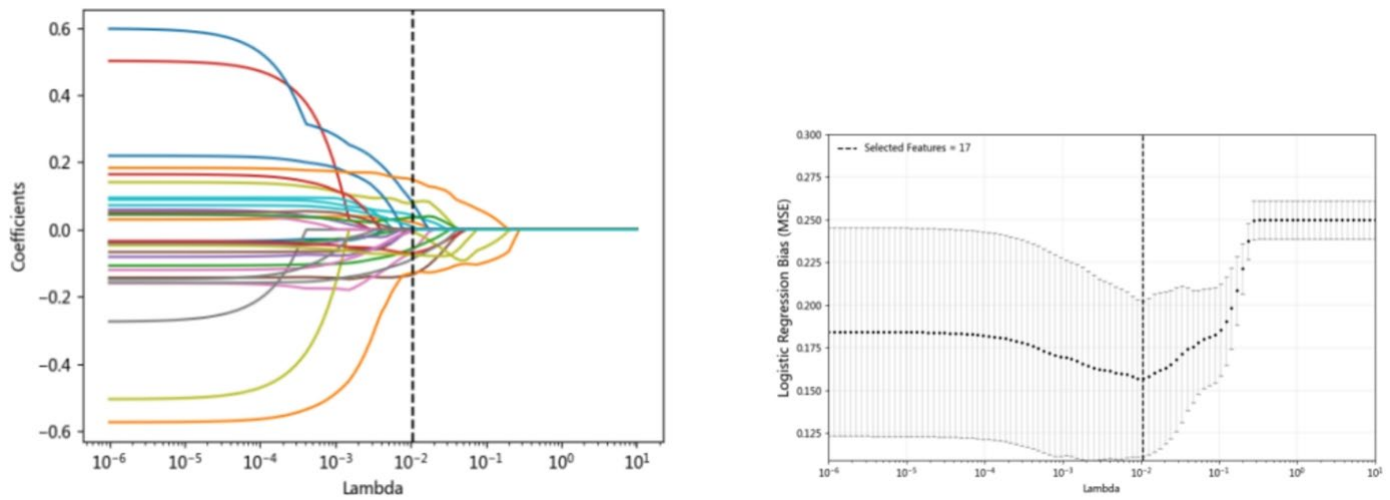
LASSO coefficient profile and optimal feature-selection pathway.

A total of 1,427 IBSI-compliant radiomic features were extracted, of which 945 features with an intraclass correlation coefficient (ICC) > 0.75 were retained. Feature dimensionality was sequentially reduced using the Mann-Whitney U test, recursive feature elimination (RFE), and finally, least absolute shrinkage and selection operator (LASSO) regression. The final set of selected features and their corresponding coefficients are presented in Table 4, and the feature-selection process is depicted in Fig. 4.

**Table 4:** Radiomic features retained for model construction and LASSO coefficients.

Feature name	Coefficient
wavelet-LLL_ngtdm_Busyness	0.55
log-sigma-3-0-mm-3D_firstorder_RootMeanSquared	-0.134
wavelet-HLH_glcm_Imc2	-0.06
log-sigma-3-0-mm-3D_glszm_ZonePercentage	-0.006
wavelet-HHH_gldm_DependenceVariance	0.041
wavelet-HLH_gldm_SmallDependenceEmphasis	0.077
original_ngtdm_Strength	-0.13
log-sigma-3-0-mm-3D_glcm_MaximumProbability	-0.055
log-sigma-3-0-mm-3D_glcm_Imc2	-0.071
logarithm_ngtdm_Busyness	-0.086
wavelet-HLH_gldm_SmallDependenceHighGrayLevelEmphasis	0.08
wavelet-HHL_glcm_InverseVariance	0.148
original_ngtdm_Busyness	0.036
log-sigma-3-0-mm-3D_firstorder_InterquartileRange	-0.001
square_glrlm_ShortRunLowGrayLevelEmphasis	-0.003
log-sigma-3-0-mm-3D_gldm_LargeDependenceLowGrayLevelEmphasis	-0.075
square_ngtdm_Strength	0.007

**Fig. 5:**
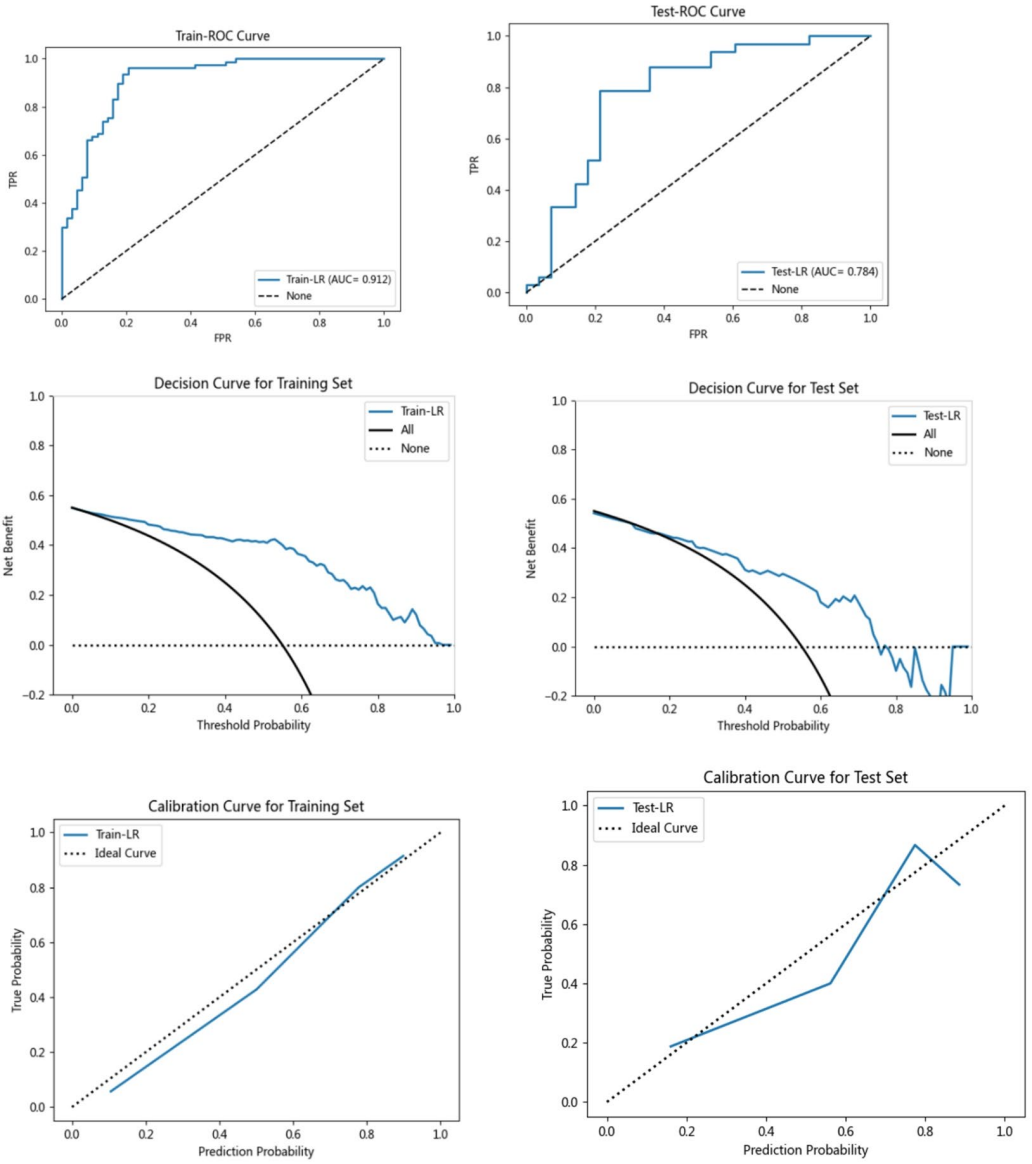
ROC, DCA, and calibration curves of the radiomics model in the training and testing cohorts.

Using the selected coefficients, a logistic regression (LR) classifier was constructed. In the training cohort, the model achieved an AUC of 0.912 (95% CI, 0.855–0.956) with an optimal cut-off value of 0.536. When applied to the independent testing cohort, the model maintained strong discriminatory performance, yielding an AUC of 0.784 (95% CI, 0.649–0.903) with an optimal cut-off of 0.699. The ROC curves, decision-curve analysis (DCA), and calibration plots for both cohorts are shown in Fig. 5.

### Deep-learning visual-classification results

Among the twelve visual-classification configurations evaluated, the “no normalization – default window width/level – 2D” model demonstrated the best overall performance. In the training cohort, this model yielded an AUC of 0.754, an accuracy of 68.9%, and an F1-score of 0.754. In the testing cohort, it achieved an accuracy of 82.5%, an AUC of 0.845, a sensitivity of 100%, and a specificity of 57.8%. The model also yielded a precision of 73.3% and an F1 score of 0.846. This performance discrepancy between the training and testing phases highlights the potential volatility associated with pure deep-learning architectures on smaller datasets.

**Fig. 6:**
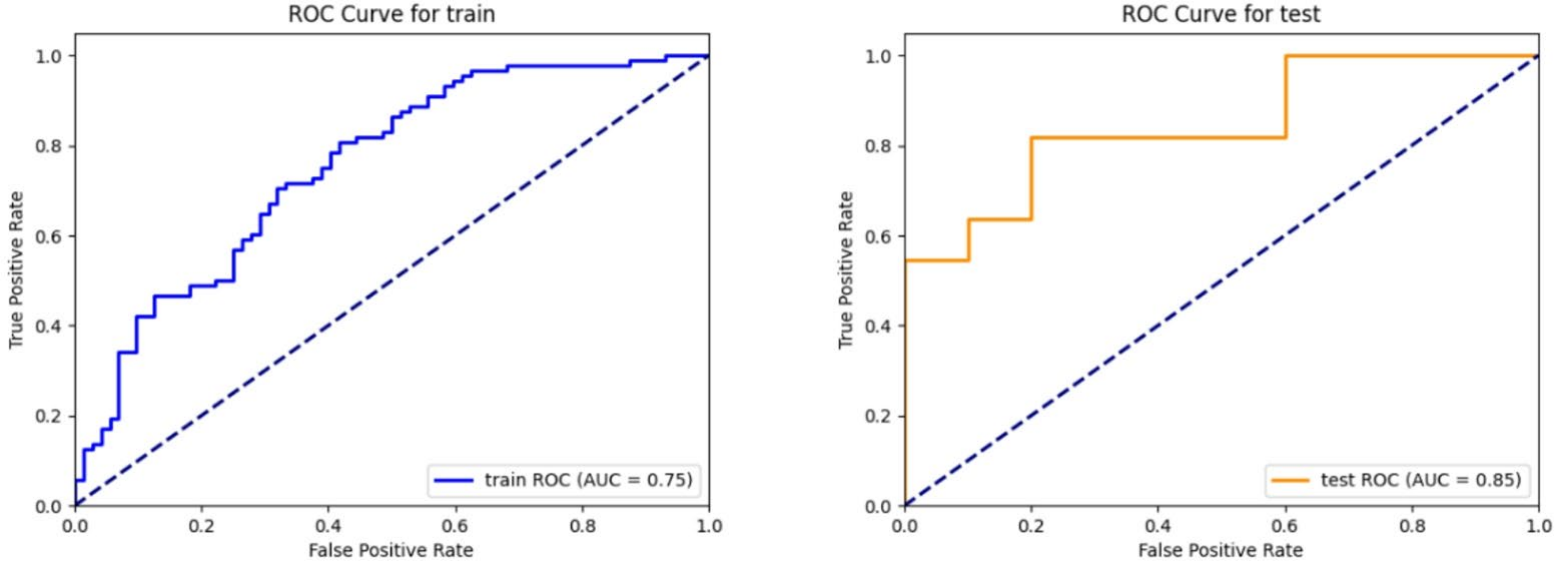
ROC curves of the best 2-D visual-classification model in the training and validation cohorts.

The 2.5D strategy, which fused axial, sagittal, and coronal slices with the largest diameters, did not outperform the optimal 2D model. Its best-performing variant, also without image normalization and at the default window setting, achieved an accuracy of 68.6%, an AUC of 0.779, a sensitivity of 81.8%, and a specificity of 52.6%. The ROC curves for the top-performing 2D visual-classification model are shown in Fig. 6.

### Fusion-model results

The predicted probabilities from the clinical, radiomics, and visual-classification models were aggregated and used to construct a stacked ensemble model. Comparative analysis demonstrated that the fusion model outperformed each individual modality. Among the five ensemble algorithms evaluated, the logistic regression (LR) model showed the highest discriminative performance, achieving an AUC of 0.936 (95% CI: 0.895–0.971) in the training cohort and 0.823 (95% CI: 0.699–0.934) in the testing cohort. In the testing set, the LR model yielded an accuracy of 0.803, a precision of 0.816, and an F1 score of 0.797.

**Fig. 7:**
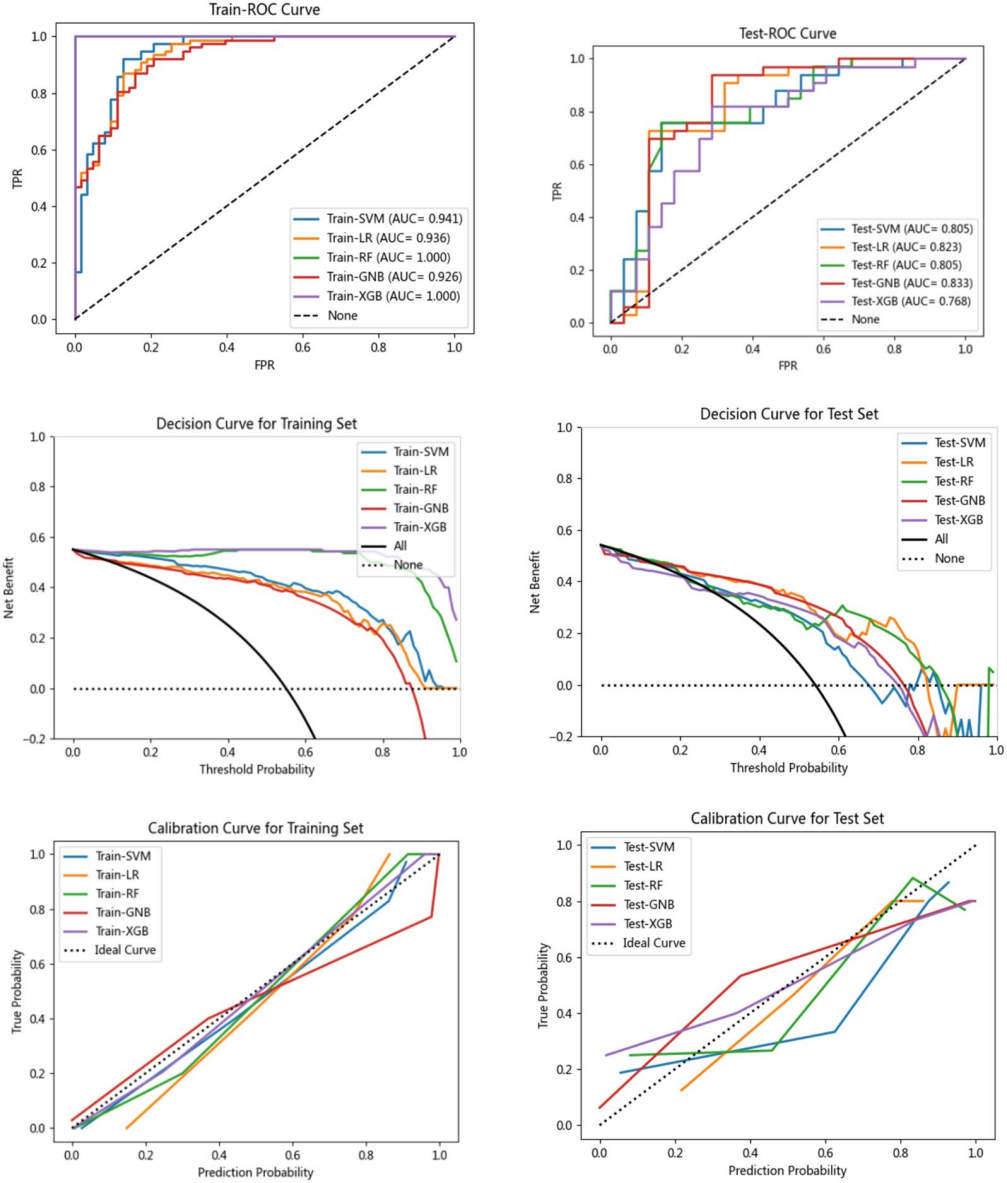
ROC, DCA, and calibration curves of the stacked-ensemble model in the training and validation cohorts.

For comparison, the AUCs of the support vector machine (SVM), Gaussian Naive Bayes (GNB), random forest (RF), and extreme gradient boosting (XGB) models in the testing cohort were 0.805, 0.833, 0.805, and 0.768, respectively. The ROC curves, decision-curve analyses (DCA), and calibration plots for the fusion model are shown in Fig. 7.

### Incremental value of multi-modal integration

To evaluate the incremental value of multi-modal integration, a head-to-head comparison was performed between the Stacked-Ensemble Model and individual modalities (Clinical, Radiomics, and Deep Learning). As summarized in Table 5, the ensemble model demonstrated superior stability and robust performance across both cohorts. While the individual Deep-Learning (DL) model achieved a high AUC of 0.845 in the testing set, it exhibited a lower training performance (0.754), indicating higher volatility compared to the ensemble framework (Test AUC: 0.823; Training AUC: 0.936). The integration of clinical, radiomic, and deep-learning features yielded more balanced performance metrics, effectively reducing the performance gap between the derivation and evaluation phases.

**Table 5:** Performance comparison of individual models and the Stacked-Ensemble Model.

Model	Cohort	Accuracy	AUC	Precision	Recall	F1-Score
Stacked-Ensemble	Training	0.864	0.936	0.876	0.855	0.86
(Combined)	Testing	0.803	0.823	0.816	0.794	0.797
Clinical	Training	0.829	0.902	0.836	0.82	0.823
	Testing	0.754	0.805	0.767	0.743	0.744
Radiomics	Training	0.864	0.912	0.881	0.854	0.859
	Testing	0.754	0.784	0.767	0.743	0.744
Deep-Learning (DL)	Training	0.689	0.754	0.655	0.886	0.754
	Testing	0.825	0.845	0.733	1	0.846

## Discussion

By integrating clinical variables, radiomic features, deep-learning visual outputs, and serum autoantibody profiles, a multimodal stacked-ensemble model was constructed to differentiate benign from malignant solitary pulmonary nodules (SPNs). Compared with conventional single-modality approaches, the fusion model based on the logistic regression (LR) meta-learner achieved an AUC of 0.823, an accuracy of 0.803, a recall of 0.794, and an F1 score of 0.797 in the testing cohort. Decision-curve analysis demonstrated consistent net clinical benefit across a wide range of threshold probabilities. These results underscore the necessity of a multi-modal approach, aligning with the conclusions of Ma et al. (2023)^20^ and Shi et al. (2025)^21^. The integration of high-dimensional radiomic features and abstract DL representations with traditional clinical benchmarks effectively overcomes the limitations of single-modality assessments. By reconciling systemic biological immune signatures with localized voxel patterns, our framework provides a more reliable tool for individualized risk stratification and clinical decision-making^20,21^.

It should be noted that a direct head-to-head comparison between our model and current clinical standards, such as PET/CT or the Brock model, was not performed. In real-world clinical practice, PET/CT is often bypassed for smaller nodules (e.g., < 10 mm) and subsolid nodules due to its limited diagnostic sensitivity, high costs, and radiation exposure. While established tools like the Brock model offer robust risk estimates based primarily on macro-clinical features, our framework functions as an objective ‘digital biopsy’ by extracting high-dimensional features that capture sub-visual nodule heterogeneity. Our baseline ‘Clinical Model’ already integrates traditional risk factors along with seven serum autoantibodies, achieving a high AUC of 0.805. The superior performance of the final stacked-ensemble model demonstrates that integrating multi-modal data provides synergistic information that exceeds the diagnostic power of conventional clinical benchmarks, offering a quantitative ‘second opinion’ to reduce diagnostic uncertainty.

Although prior investigations have yielded promising advances in SPN characterization, notable constraints persist. Liu et al.^22^ constructed a radiomics nomogram from 875 cases that combined age and CT features, attaining an AUC of 0.809 in the validation cohort; however, the clinical variables were rudimentary, potentially overlooking key tumor-microenvironment signals, and the fusion strategy treated modalities in isolation. In a prospective study, Baeza et al.^23^ merged pulmonary-function metrics with deep-learning image features via a Bayesian framework, yet their cohort comprised only 97 nodules and lacked biomarker confirmation, limiting external applicability. Mao et al.^24^ recently employed GPT-4o to analyze longitudinal CT series from 647 patients and showed that large-language models can dynamically appraise nodule evolution (malignancy-prediction accuracy 0.88), but the purely imaging-driven paradigm lacked multimodal synthesis and suffered from “black-box” opacity. Saied et al.^25^ systematically compared conventional machine learning with deep learning for nodule classification, reporting an AUROC of 0.96 for DenseNet-121, yet their work was confined to a single imaging modality and did not explore the complementary value of clinical or molecular data. Collectively, these studies highlight the progress made, but also reveal persistent gaps in cross-modal integration and biological interpretability, which our multi-modal stacked-ensemble framework effectively addresses.

Compared with previous studies, the present work introduces several methodological innovations designed to ensure robust generalization despite a modest sample size. First, by integrating seven serum autoantibodies (e.g., p53, GAGE7) with radiomic features and deep-learning visual classifications, malignant nodules are characterized at both the molecular and morphological levels. The resulting multimodal stacked-ensemble model achieved an AUC of 0.823 in the testing cohort, surpassing the performance of the hybrid model proposed by Baeza et al. (AUC = 0.80) and the radiomics nomogram developed by Liu et al. (AUC = 0.809). Second, the Swin-Transformer–based visual classifier reached an accuracy of 82.5% using raw, non-normalized CT data. To mitigate overfitting, this architecture employed structural regularization—substituting original stages with a lightweight FusionCNN and incorporating DropPath and online data augmentation—forcing the model to learn stable morphological patterns rather than memorizing training noise. Although this is lower than the 90.39% accuracy reported for DenseNet-121 by Saied et al., the integration of clinical, imaging, and serological features in the present model yielded superior net clinical benefit across a broad decision-threshold range (5%–90%), as demonstrated by decision-curve analysis. Third, to achieve multi-stage feature sparsity, LASSO regression identified several radiomic features with strong associations to malignancy (e.g., *wavelet-LLL_ngtdm_Busyness*), and statistical analysis revealed that serum PGP9.5 positivity was significantly more frequent in the malignant group (*p* < 0.05). These variables offer biologically interpretable insights that are not available in the purely image-based GPT-4o framework proposed by Mao et al. (2023).

Previous research has shown that malignant tumors—especially early-stage lung cancer—can trigger measurable tumor-associated autoantibodies^14^. Such assays are convenient, inexpensive and analytically stable, and they improve sensitivity for subsolid micronodules^26^. Importantly, adding autoantibody testing did not materially reduce specificity; it did not merely raise false-positive rates but truly captured subtle molecular abnormalities in high-risk individuals.

Poor model generalization remains the principal barrier to the clinical deployment of AI algorithms^27^. External validation across multiple centers, diverse scanners and heterogeneous pathological compositions is therefore essential. Although a geographically distinct external cohort was not available, evaluating the model across three global CT vendors (TOSHIBA, UIH, and GE) subjected it to a rigorous technical evaluation that mimics the heterogeneity of true external validation. In our independent multicenter test cohort, the stacked model retained an AUC exceeding 0.80 and showed favorable calibration, with no ‘performance cliff’ observed compared to the training phase, underscoring its empirical stability. Methodologically, we adopted a probability-stacking strategy rather than conventional early data-level fusion or late voting: the predicted probabilities from each unimodal model were repurposed as new features for a reconstructed ensemble. This approach offers two advantages: (i) it mitigates the risk of overfitting that can arise from directly concatenating high-dimensional radiomic features with clinical data, and (ii) it allows each unimodal model to be optimized independently, enhancing overall flexibility. Moreover, the introduction of the lightweight FusionCNN and large-kernel QJBlock modules alleviated the Swin-Transformer’s limited capacity to capture irregular nodule shapes within local windows^28^. Finally, the key features selected via LASSO not only render the model more “white-box” and clinically interpretable but also provide actionable guidance for practice and a foundation for future mechanistic research.

Our multi-modal stacked model is envisioned as a secondary screening tool within the standard radiology workflow. To facilitate clinical translation, we envision a four-stage implementation pathway for our integrated model within existing diagnostic frameworks: (1) Data Harmonization, where CT scans undergo voxel-level intensity normalization to ensure cross-scanner stability; (2) Automated Characterization, where the pipeline performs parallel extraction of deep-learning, radiomic, and clinical features; (3) Risk Stratification, where the stacked model outputs a final integrated probability score; and (4) Management Triage, where the score assists clinicians in stratifying SPNs ($\:\le\:30$ mm) into ‘low-risk’ (conservative surveillance) or ‘high-risk’ (prompt biopsy or resection) categories. On standard clinical workstations, the end-to-end computational time is less than 10 s, facilitating real-time decision support. From a cost-effectiveness perspective, the model’s high specificity serves as a ‘gatekeeper’ for invasive procedures, significantly reducing medical costs and potential complications associated with unnecessary biopsies or resections. By acting as an objective ‘second opinion,’ this pathway aims to reduce the medical burden for benign nodules while ensuring timely intervention for malignant cases.

Several limitations of this study should be acknowledged. First, the total cohort size (*n* = 201) is relatively modest, and the retrospective design precluded an a priori sample size calculation. However, the post-hoc 95% CIs for all models remained significantly above the random-chance threshold, confirming sufficient statistical power for diagnostic evaluation. Although the data were sourced from a single medical consortium, the inclusion of three centers with distinct clinical roles (e.g., geriatric vs. general hospitals) and different CT vendors (TOSHIBA, UIH, and GE) provided substantial technical and demographic heterogeneity. Consequently, the model was validated on an internal multi-center test set rather than a geographically independent external cohort. While the high technical heterogeneity across vendors provides a degree of robust verification, the absence of true external validation limits the immediate generalization of our findings to diverse populations. Therefore, we formally recommend that future prospective studies utilize datasets from independent medical networks across different regions to further confirm the model’s clinical utility and global generalizability.

Furthermore, the retrospective nature meant that CT acquisition parameters were not perfectly uniform across centers, potentially introducing batch effects. In future work, we intend to implement standardized imaging protocols aligned with the Image Biomarker Standardization Initiative (IBSI) and explore advanced harmonization methods, such as ComBat or deep-learning-based domain adaptation. Additionally, although we ensured high internal consistency by utilizing a centralized laboratory for autoantibody detection, potential variability across different assay platforms remains a constraint. Future prospective studies with larger, geographically independent cohorts and cross-platform validation are necessary to fully establish the global generalization and clinical utility of our multimodal stacked model.

## Conclusion

A multimodal stacked-ensemble model was developed by combining clinical variables, radiomic features, deep-learning image classifications, and serum autoantibodies to differentiate benign from malignant solitary pulmonary nodules (SPNs) while mitigating the “black-box” limitations of pure deep learning architectures. In the independent testing cohort, the model achieved an AUC of 0.823, outperforming all single-modality approaches. This integrative framework demonstrates potential as a more efficient, robust, and interpretable auxiliary tool for the clinical evaluation of SPN malignancy.

## Supplementary Information

Below is the link to the electronic supplementary material.

Supplementary Material 1

## Data Availability

The data that support the findings of this study are available from the corresponding author upon reasonable request.
