# Supplementary material for: Stacked CT radiomics, deep learning and clinical feature models for differentiating benign and malignant solitary pulmonary nodules
Source: Sci Rep. 2026 Apr 28;16:19680. doi: 10.1038/s41598-026-49720-8 (PMC13315583; doi:10.1038/s41598-026-49720-8)
Supplement: Supplementary file 1 — Supplementary Material 1 [file 41598_2026_49720_MOESM1_ESM.docx]

**Supplementary Table S1.** Detailed CT acquisition parameters across the three participating centers.

| Parameters | Huabei Petroleum Geriatric Hospital (TOSHIBA Alexion CT) | Huabei Petroleum Oilfield Construction Hospital (UIH uCT550 CT) | Huabei Petroleum Administration Bureau General Hospital (GE Revolution CT) | Huabei Petroleum Administration Bureau General Hospital (GE LightSpeed VCT) |
| --- | --- | --- | --- | --- |
| Tube Voltage (kVp) | 120 | 120 | 120 | 120 |
| Tube Current (mA) | 220 | Adjustment | Adjustment | Adjustment |
| Pitch | 1.1:1 | 1.025:1 | 0.984:1 | 0.985:1 |
| Collimation | 0.625mm × 16 | 0.55mm × 40 | 0.625mm × 64 | 0.625mm × 64 |
| Gantry Rotation Time (s/rot) | 1.2 | 0.8 | 0.6 | 0.6 |
| SFOV (cm) | 50 | 50 | 50 | 50 |
| Slice Thickness (mm) | 1 | 1.25 | 0.625~1.25 | 0.625~1.25 |
| Slice Interval (mm) | 1 | 1.25 | 0.625~1.25 | 0.625~1.25 |
| Reconstruction Algorithm | Lung Reconstruction Algorithm | Lung Reconstruction Algorithm | Lung Reconstruction Algorithm | Lung Reconstruction Algorithm |
| Image Matrix | 512 × 512 | 512 × 512 | 512 × 512 | 512 × 512 |

**Legend:** kVp: kilovolt peak; mA: milliampere; SFOV: Scan Field of View. Note: Tube current (mA) was automatically adjusted for UIH and GE scanners based on patient size. All images were reconstructed using a uniform Lung Reconstruction Algorithm to minimize source-level variability.
